# Utilisation of Amaranth and Finger Millet as Ingredients in Wheat Dough and Bread for Increased Agro-Food Biodiversity

**DOI:** 10.3390/foods11070911

**Published:** 2022-03-22

**Authors:** Calvin Onyango, Susan Karenya Luvitaa, Kibet Lagat, Alexandra Hüsken, Inga Smit, Marcus Schmidt

**Affiliations:** 1 Food Technology Division, Kenya Industrial Research and Development Institute, Nairobi P.O. Box 30650-00100, Kenya; skalenya@gmail.com (S.K.L.); jlagat78@gmail.com (K.L.); 2 Department of Safety and Quality of Cereals, Max Rubner-Institut (MRI), Federal Research Institute of Nutrition and Food, Schuetzenberg 12, 32756 Detmold, Germany; alexandra.huesken@mri.bund.de (A.H.); inga.smit@mri.bund.de (I.S.); marcus.schmidt@mri.bund.de (M.S.)

**Keywords:** amaranth, bread, dough, finger millet, wheat

## Abstract

Amaranth and finger millet are important food security crops in Africa but show poor bread making ability, even in composite wheat breads. Malting and steaming are promising approaches to improve composite bread quality, which have not been fully explored yet. Therefore, in this study, wheat was blended with native, steamed or malted finger millet or amaranth in the ratio of 70:30. Wheat/native amaranth (WHE-NAM) and wheat/malted amaranth (WHE-MAM) had longer dough development times and higher dough stabilities, water absorption capacities and farinograph quality numbers than wheat/steamed amaranth (WHE-SAM), wheat/native finger millet (WHE-NFM), wheat/steamed finger millet (WHE-SFM) or wheat/malted finger millet (WHE-MFM). The WHE-NAM and WHE-MAM breads had lower crumb firmness and chewiness, higher resilience and cohesiveness and lighter colours than WHE-NFM, WHE-SFM and WHE-MFM. Starch and protein digestibility of composite breads were not different (*p* > 0.05) from each other and ranged between 95–98% and 83–91%, respectively. Composite breads had higher ash (1.9–2.5 g/100 g), dietary fibre (5.7–7.1 g/100 g), phenolic acid (60–122 mg/100 g) and phytate contents (551–669 mg/100 g) than wheat bread (ash 1.6 g/100 g; dietary fibre 4.5 g/100 g; phenolic acids 59 mg/100 g; phytate 170 mg/100 g). The WHE-NAM and WHE-MAM breads possessed the best crumb texture and nutritional profile among the composite breads.

## 1. Introduction

Finger millet (*Eleusine coracana*) and amaranth (*Amaranthus cruentus*) are important food security crops in sub-Saharan Africa because they are high-yielding crops, even under adverse agro-ecological environments. In addition, they are valuable sources of energy and proteins. However, they have limited utilization in industrial food product development. One technique to increase consumption of these crops is by incorporating them in ready-to-eat foods such as bread [1,2,3].

Partial substitution of wheat with non-wheat flours improves the nutritional quality of bread, promotes consumption of underutilized crops and increases sensory diversity of bread [1,2,3]. Unfortunately, composite bread has lower volume and harder crumb compared to wheat bread because the non-wheat flour dilutes gluten and the gluten matrix cannot develop properly due to interference by non-wheat flour constituents, such as dietary fibre [3,4,5]. These envelop the gluten proteins, limiting the formation of a network.

The major strategies used to manage the negative effects of non-wheat flours in composite dough and bread include using wheat flour with high protein content [5,6] and limiting the amount of non-wheat flour to about 30–40% *w*/*w* [2,7,8]. In addition, vital gluten, ascorbic acid, emulsifiers, enzymes and hydrocolloids [2,9,10] can be added to composite dough to compensate for gluten dilution and support development of the gluten matrix. Amaranth albumin proteins also improve rheological properties of dough by interacting with gluten proteins through disulphide bonds [11]. Bread with added amaranth albumin proteins has higher volume and better crumb texture compared to wheat bread [12].

The quality of composite bread can also be improved by modifying non-wheat flours prior to blending them with wheat. Guardianelli et al. [13] found that germinated amaranth improves elasticity and viscosity of composite dough, while Mlakar et al. [14] found that composite dough made from wheat and amaranth whole-grain flour had higher stability and strength compared to wheat dough. However, these two studies did not report on the impact of amaranth flours on the quality of composite bread. Tosi et al. [15] reported that low levels (4% flour-weight-basis) of defatted hyperproteic amaranth flour has no negative impact on specific volume of bread. Composite dough containing extruded finger millet is less firm and more extensible than dough containing unextruded finger millet [16]. The resulting bread has higher volume and better crumb texture than bread containing unextruded finger millet [16]. Other examples of modified cereals that have been used to improve volume and crumb texture of composite bread are germinated brown rice flour [17] and fermented sorghum [18]. There is still a lack of studies on the impact of finger millet on the quality of composite bread. It is a native African crop with remarkable resilience against heat and drought stress combined with high storage stability and good nutritional values. Thus, incorporation of finger millet into various food products should be studied more intensely. Onyango et al. [1] reported that composite bread containing hydrothermally treated finger millet had softer crumb and higher volume compared to composite bread containing native finger millet. The use of steaming or malting to improve the bread making abilities of amaranth and finger millet has not been studied yet. However, it was found that composite bread made with boiled malt flour has better crumb texture and lower degree of staling compared with composite bread made with native sorghum flour [19]. Based on these results, malting and steaming appear as promising tools to improve the quality of composite wheat bread. Hence, it was the aim of this study to close this knowledge gap by comparing the effect of steamed or malted finger millet and amaranth on the rheological properties of dough and physico-chemical quality of bread. These results are an important contribution towards an optimized utilisation of the nutritionally and economically valuable crops amaranth and finger millet.

## 2. Materials and Method

### 2.1. Modification of Finger Millet

Native finger millet (NFM) and amaranth (NAM) grains were purchased in Busia County, Kenya. Steamed finger millet (SFM) and amaranth (SAM) were prepared by washing the grains before steeping them in water (1:5 *w*/*v*) for 24 h at 24 ± 1 °C. After steeping, excess water was drained through a filter cloth before the grains were placed in a stainless-steel container, covered with an aluminium sheet and steamed in an autoclave (Biobase Co., Ltd., Shandong, China) at 100 °C for 20 min. Malted finger millet (MFM) and amaranth (MAM) were prepared using a modified method by Hugo et al. [19]. The grains were washed and steeped as described for SAM and SFM and excess water was removed. The grains were then spread on woven polypropylene cloth, which was spread on perforated aluminium tray for 48 h at 24 ± 1 °C. The tray was loosely covered with another woven polypropylene cloth. Twice daily, water was sprinkled on the grains before they were gently mixed. After malting, the grains were steamed, as described for SAM and SFM. The steamed and malted grains were dried to 12 ± 2% moisture content in an electric oven (Memmert GmbH + Co. KG., Schwabach, Germany) set at 60 °C over a period of 48 h. The grains were milled using a Bauermeister universal turbo laboratory (UTL) grinder fitted with 500 μm sieve (Bauermeister Maschinenfabrik GmbH, Hamburg-Altona, Germany).

### 2.2. Characterization of Flours

Protein (N × 5.75 for wheat; N × 6.25 for finger millet and amaranth, respectively), lipid and ash contents of flours were determined on dry-weight basis according to ICC standard methods No., 105/2, 136 and 104/1, respectively [20,21]. Total and digestible starch and phytate contents were measured using K-RAPRS and K-PHYT kits, respectively (Megazyme Int. Ireland Ltd., Wicklow, Ireland). Soluble, insoluble and total dietary fibre contents were measured using K-TDFR-100A kit from Megazyme Int. (Wicklow, Ireland).

Free soluble sugars were determined following the procedure described by Schmidt and Sciurba [22] with some modifications. In brief, 1.00 ± 0.01 g of sample was combined with 2.0 mL methanol and homogenized. After adding 20 mL of deionized water (80 °C), the suspension was homogenized by ultrasonication (BANDELIN Sonoplus, Berlin, Germany) for 2 × 15 s at room temperature. After centrifugation (5 min, 1700× *g*, 20 °C), the supernatant was transferred to a 100 mL volumetric flask and the hot water extraction was repeated twice. Proteins were removed from the combined supernatants by adding 500 µL of Carrez I (15% *w*/*v*) and Carrez II (32% *w*/*v*), respectively. After adjusting to volume, solids were removed by centrifugation (10 min, 3000× *g*, 20 °C) and the supernatant was filtered (0.45 µm) into a HPIC vial. For analysis of the sample extracts, high-performance anion exchange chromatography (HPAEC, Dionex ICS 5000+, Sunnyvale, CA, USA) with pulsed amperometric detection (PAD) was used. The system was equipped with a CarboPac PA 1 guard column and a CarboPac PA1 analytical column (4 × 250 mm), both operated at 25 °C. The mobile phase consisted of (A) 100 mM sodium hydroxide solution and (B) 600 mM sodium acetate in 100 mM sodium hydroxide. Eluents were degassed and stored under helium atmosphere. The following gradient program was applied for separation: 0–40 min 100% A, 40–55 min linear increase of B from 0 to 100%, 55–70 min 100% A. The injection volume was 25 µL, the flow rate was set to 1.0 mL/min for a total run time of 70 min. For calibration, analytical standards of raffinose, maltose, sucrose, glucose, fructose, sorbitol and mannitol were used in various dilutions, between 0.1 and 30 mg/100 mL. All calibrations were found to be linear in the respective calibration range (R^2^ > 0.99). Limit of detection (LOD) and limit of quantification (LOQ) were set for a signal to noise ratio of 3 and 10, respectively.

Determination of the total arabinoxylan content was based on the method of Houben et al. [23]. The sample (0.05 ± 0.001 g) was suspended in 1 mL of deionized water and 2 mL of hydrochloric acid (4 M). The homogenized suspensions were incubated for 90 min at 100 °C and homogenized every 10 min. After cooling to room temperature, mixtures were neutralized with 2 mL sodium hydroxide (4 M). Subsequently, 1 mL of Tris buffer (0.2 M, pH = 7.6) and 1 mL of glucose oxidase-catalase solution from Megazyme Int. (Wicklow, Ireland) were added and homogenized. The suspension was incubated for 60 min at 30 °C, centrifuged (10 min, 3500 g, 20 °C) and the supernatant filtered (0.45 µm) into a HPIC vial. Analysis was carried out similarly to the free soluble sugars described above, with the following modifications. The mobile phase consisted of (A) 20 mM sodium hydroxide solution and (B) 600 mM sodium acetate in 100 mM sodium hydroxide. For analysis, the following gradient program was used: 0–30 min 100% A, 30–45 min linear increase of B from 0 to 100%, 45–60 min 100% A. Total run time was 60 min. For calibration, analytical standards of arabinose and xylose were used in various dilutions, between 0.1 and 30 mg/100 mL.

Characterization of process-induced changes in arabinoxylan molar mass was carried out using gel permeation chromatography (GPC). For extraction, 200 mg of ground sample was suspended in 10 mL of sodium nitrate solution (0.1 M), containing 0.02% sodium azide, and incubated for 2.5 h at 90 °C. Subsequently, the mixtures were cooled to 50 °C and after addition of 200 µL lichenase solution (10 U) incubated for 1 h at 50 °C. After addition of 10 µL α-amylase solution (5 mg/mL in 3.6 mM CaCl_2_) and incubation at 37 °C for 1 h, 50 µL of Carrez I (15% *w*/*v*) and Carrez II (32% *w*/*v*) were added, the mixture was centrifuged and the supernatant filtered (0.45 µm) into a GPC vial. Measurement was carried out using the GPCmax gel permeation chromatography system (Malvern Panalytical, UK). For separation, a A6000M (300 × 8 mm), Aq GPC/SEC double column, equipped with an AGuard pre-column (50 × 6 mm) was used. The measurement was done isocratic using 0.1 M sodium nitrate solution, containing 0.02% sodium azide, at a flow rate of 1.0 mL/min. The column temperature was held at 30 °C, the run time was 35 min and the injection volume was 100 µL. Discrete pullulan molar mass standards were used for conventional calibration, to determine arabinoxylan molar mass. In vitro protein digestibility (IVPD) and free phenolic compounds were determined as previously reported by Onyango et al. [1].

### 2.3. Properties of Flours and Doughs

Composite flour was prepared from baker’s wheat (WHE) flour (Unga Ltd., Nairobi, Kenya) and native, steamed or malted finger millet (WHE-NFM, WHE-SFM, WHE-MFM) or amaranth (WHE-NAM, WHE-SAM, WHE-MAM) at a ratio of 70:30. The α-amylase activity was measured using K-CERA kit from Megazyme Int. (Wicklow, Ireland). Dough properties were evaluated using a Farinograph-AT and an Extensograph-E (Brabender GmbH & Co. KG., Duisburg, Germany) according to ICC standard methods No. 115/1 and ICC No. 114/1, respectively [20,21].

### 2.4. Bread Making and Evaluation of Physical and Textural Properties of Breads

Breads were made by the straight dough method, as previously described by Onyango et al. [1] from WHE (control) or composite flours (wheat: non-wheat flour 70:30). The remaining baking ingredients were: sugar (2% flour-weight-basis, fwb, Kibos Sugar & Allied Industries, Kisumu, Kenya), active dry yeast (1% fwb, Angel Yeast Co., Ltd., Beni Suef, Egypt), baker’s fat (1% fwb, Bidco Africa Ltd., Nairobi, Kenya) and salt (1% fwb, Kensalt Ltd., Nairobi, Kenya). The farinograph water absorption capacities of the flours were adjusted to reach consistencies of 500 farinograph units (FU). Farinograph water absorption capacity of WHE, WHE-NFM, WHE-SFM and WHE-MFM was 59, 57, 60 and 60%, respectively. Farinograph water absorption capacity of WHE-NAM, WHE-SAM and WHE-MAM was 61, 63 and 62%, respectively. The ingredients were combined and mixed at low speed using a spiral dough hook for 1 min and further kneaded for 5 min at medium speed in a SP22HI planetary mixer (SPAR Food Machinery Mfg. Co. Ltd., Taichung Hsien, Taiwan). After resting for 15 min the dough was divided into 400 g pieces before it was manually rounded and rested for another 15 min. The dough was molded manually, loaded into baking tins (dimensions: L × W × H: 205 × 105 × 70 mm) and proofed for 60 min at 35 °C and 80% relative humidity in a proofing cabinet (National Mfg. Co., Lincoln, NE, USA). After proofing, the tins were placed in a rotary oven (National Mfg. Co., Lincoln, NE, USA) set at 200 °C and baked for 35 min. After de-panning, the loaves were kept in paper bags stored in an incubator at 25 °C for 22 h before further analysis. Bread weight, volume and specific volume were determined as described by Onyango et al. [1]. Briefly, bread was weighed on a Shimadzu analytical balance (Shimadzu Corporation, Kyoto, Japan). Bread volume was determined by displacement of finger millet in a 10 L stainless-steel container. Specific volume was calculated by dividing bread volume with weight. Change in crumb lightness (ΔL* = L*_wheat bread_ − L*_composite bread_), was measured using a Chroma Meter CR-10 (Konica Minolta, Sakai, Japan), in order to determine the impact of the pre-treatments (malting and steaming) of finger millet or amaranth on crumb appearance. Texture Profile Analysis of bread crumb was measured as previously reported by Onyango et al. [1]. Briefly described, 20 mm thick slices of bread were compressed using a 75 mm diameter aluminium cylinder probe (P/75) which was attached to a TA-XT plus Texture Analyser (Stable Micro Systems, Surrey, UK). The operating variables of the probe were calibration height (40 mm), pre-test speed (1 mms^−1^), test-speed (5 mms^−1^), post-test speed (5 mms^−1^), penetration distance (10 mm), trigger force (0.05 N).

### 2.5. Nutrient Qualities of Breads

Bread was dried at 40 °C and milled using a Bauermeister universal turbo laboratory (UTL) grinder fitted with 500 μm sieve (Bauermeister Maschinenfabrik GmbH, Hamburg-Altona, Germany). Total starch, digestible starch, protein, IVPD, lipid, dietary fibre, ash, phytate, arabinoxylan, free soluble sugars and total phenol contents were determined as described in Section 2.2.

### 2.6. Experimental Design and Statistical Data Analysis

All experimental analyses were done in duplicate or triplicate and the results were reported as mean ± standard deviation. The effect of flour type on the properties of flour, dough and bread was evaluated in a single factor experimental design. The data obtained were subjected to one-way analysis of variance. Tukey’s Test at a confidence level of 95% was used to evaluate differences in treatment means. The data were analyzed using Minitab 17 statistics software (Minitab Inc., State College, PA, USA).

## 3. Results and Discussion

### 3.1. Characterization of Flours

The distribution of free sugars and sugar alcohols in native and modified flours is shown in Table 1. Disaccharides were the major sugars in wheat and native finger millet, whereas disaccharides and trisaccharides were the major sugars in native amaranth. These results agree with those of Dharmaraj and Malleshi [24] who found that glucose, fructose, sucrose and maltose are the main sugars in finger millet; and Becker et al. [25] who reported that sucrose and raffinose are the major sugars in amaranth. The content of free sugars (i.e., monosaccharides, disaccharides and trisaccharides) decreased from 851 to 750 mg/100 g and from 1609 to 1326 mg/100 g in finger millet and amaranth, respectively, after steaming. The loss of free sugars in steamed finger millet is due to leaching during steeping and Maillard reactions during steaming [24]. The content of free sugars increased from 851 to 1311 mg/100 g and from 1609 to 2013 mg/100 g in finger millet and amaranth, respectively, after malting. These changes were attributed to enzymatic hydrolysis of starch into sugars during germination. Starch content declined by 4 and 16% for finger millet and amaranth, respectively, whereas monosaccharide content of the grains increased almost 20 times after malting. The levels of sugar alcohols in all the grains were ≤63 mg/100 g and decreased for finger millet but increased for amaranth after the grains were steamed or malted.

Amaranth is a dicotyledonous plant that is commonly referred to as a pseudocereal because its chemical composition and techno-functionality resemble those of true cereals. However, the starch content of amaranth seeds is lower than that of true cereals, such as wheat and finger millet (Table 1). Amaranth had higher (*p* < 0.05) digestible starch content than wheat or finger millet. Starch granule size is an important factor in determining the rate of starch digestion and, usually, smaller granules are digested faster than larger granules. In this case, the smaller amaranth starch granules (1–2 µm) have higher surface area to volume ratio for enzymatic hydrolysis than the larger finger millet (4–12 µm) and wheat starch (15–35 µm) granules [26].

Finger millet and amaranth had 3 to 5 times more insoluble dietary fibre but 1 to 2 times less soluble dietary fibre than wheat (Table 1). Overall, finger millet and amaranth had 2 to 5 times more total dietary fibre than wheat. The low insoluble dietary fibre content in refined wheat can be attributed to separation of the starchy endosperm from the bran during milling. By contrast, finger millet and amaranth had high insoluble dietary fibre contents, since bran was not separated from the endosperm during milling. Arabinoxylan content declined when finger millet was steamed but increased when it was malted or when amaranth was steamed or malted. The increase in arabinoxylan content in malted grains may be because steeping and germination softened the cell wall tissues resulting in improved extractability [27]. Despite the changes in arabinoxylan contents of the grains after steaming or malting, there was no corresponding change in their average molecular weights (Table 1).

The net change in protein content of grains after malting is influenced by the balance between leached water-soluble peptides versus starch breakdown via respiration. Thus, protein content of malted finger millet may have declined because the loss of water-soluble peptides exceeded the degree of starch degradation. In contrast, protein content in malted amaranth increased because starch breakdown exceeded the leaching of water-soluble peptides. Although the contents of water-soluble peptides were not determined, changes in starch contents due to germination vary substantially and are likely to have an impact on the protein contents of the grains. Amaranth lost a greater amount of starch (16%) after germination than finger millet (4%). The IVPD of finger millet and amaranth were between 87 and 88% and did not change (*p* > 0.05) after malting or steaming. These results differ from published literature, which indicate that IVPD of finger millet and amaranth increase after malting or steaming [24,28,29]. The native materials had inherently high IVPD contents, which did not further increase after malting or steaming. Other authors have reported lower IVPD values in native finger millet and amaranth and substantial increases after steaming or germination. Dharmaraj and Malleshi [24] reported that IVPD in finger millet increased from 79 to 91% after steaming, whereas Hejazi and Orsat [29] found that it increased from 74 to 92% after germination. Olawoye and Gbadamosi [28] found that IVPD increased from 36% in native amaranth to 58 and 65% after steaming and germination, respectively.

The lipid, ash, phytate and phenolic acid contents of the grains reflected their different botanical origins and effect of processing (Table 1). Amaranth had higher (*p* < 0.05) lipid content than wheat or finger millet. Finger millet and amaranth had higher (*p* < 0.05) ash and phytate contents than refined wheat. Phytate content of finger millet did not change (*p* > 0.05) whereas that of amaranth increased after steaming or malting. Generally, phytate content decreases after germination [29] due to synthesis or activation of endogenous phytases, which hydrolyse myo-inositol 1,2,3,4,5,6-hexakisphosphate (IP6) into lower inositol phosphates such as IP5, IP4, IP3, IP2, IP1 and myo-inositol. However, phytate content may also increase after malting [30] if the lower forms of phytic acid are co-eluted as total phytic acid during extraction [31]. Phenolic acid contents of native flours followed the order: amaranth < wheat < finger millet. In finger millet, the phenolic acid content did not change (*p* > 0.05) after steaming but increased (*p* < 0.05) after malting. Malted or steamed amaranth contained higher (*p* > 0.05) phenolic acid content than in the native grain. Phenolic acid content increases in grains after malting due to the action of esterases on phenolic acid esters linked to arabinoxylans and other non-starch polysaccharides [27].

### 3.2. Properties of Flours and Doughs

The α-amylase activities of the flours and dough properties derived from the farinograms are shown in Table 2. There were no significant differences (*p* > 0.05) in the α-amylase activities of the flours. Especially noteworthy were the low α-amylase activities of flours containing malted finger millet or amaranth. Although germination induces de novo synthesis of α-amylase in grains, the diastatic activity can be regulated or inactivated by heat treatment [19].

The water absorption capacity of wheat is determined by its protein, arabinoxylan and damaged starch contents, hardness and particle size index [32]. This value is enhanced further in composite flours with high protein or dietary fibre contents [5,15], as was noted for amaranth and finger millet. Dietary fibre has huge impact on water absorption capacity of flour because the numerous hydroxyl groups in the molecular structure of non-starch polysaccharides allow for multiple water interactions through hydrogen bonds [6,33]. In addition, the high water absorption capacity of WHE-NAM, WHE-SAM and WHE-MAM doughs could be attributed to the high water binding capacity of amaranth starch granules and albumins [12,15].

The WHE-NAM, WHE-SAM and WHE-MAM had higher dough development times than wheat or WHE-NFM, WHE-SFM and WHE-MFM doughs. Composite doughs, except WHE-MFM, had higher dough stabilities than wheat. However, prolonged mixing of doughs showed that composite doughs had higher (*p* < 0.05) degrees of softening compared to wheat (Table 2). The quantity and quality of gluten proteins determine the mixing behaviour of hydrated wheat and the rheological character of optimally mixed dough. When wheat flour is hydrated and kneaded, discrete masses of gluten protein are transformed into a continuous cohesive viscoelastic network. During kneading, dough resistance increases to an optimal state before it begins to decrease. The changes in resistance to mixing are recorded in the farinograph as dough development time, dough stability and degree of softening. The gluten protein network, formed during kneading, is responsible for retaining carbon dioxide produced during fermentation and in the initial stages of baking, thus determining bread volume and crumb structure [34]. While in wheat doughs gluten is the determining factor for dough development and stability, in composite doughs, dietary fibre [5,6,33] and proteins [11,12] of the non-wheat constituents also play an important role. Dietary fibre increases dough development time, because non-starch polysaccharides require more time to absorb water before dough reaches optimal consistency [5]. With respect to amaranth, the albumin proteins also form intermolecular disulphide bonds with wheat glutenins and produce dough with rheological character similar to glutenin polymers in wheat [11,12]. Despite the positive effects of finger millet and amaranth on the development and stability of composite doughs, prolonged mixing of these doughs eventually destroyed and weakened their gluten networks and increased their degrees of softening. The farinograph quality number was positively correlated with dough development time and stability. The WHE-NAM, WHE-SAM and WHE-MAM doughs had high farinograph quality numbers, which agreed with their long dough development times and high dough stabilities. In contrast, WHE-MFM dough had the lowest farinograph quality number, which was consistent with its short dough development time and low dough stability.

Composite doughs had lower (*p* < 0.05) energies, extensibilities and resistances to extension than wheat dough at all incubation times (Table 3). These findings are similar to those of Koletta et al. [5] and Mlakar et al. [15] and show that composite doughs were more rigid and required less work to stretch compared to wheat. The viscoelastic character of wheat is influenced by the two gluten fractions: gliadin and glutenin. Glutenin polymers form viscoelastic networks that provide strength (resistance to extension) and elasticity to dough, whereas gliadin acts as plasticizer within the glutenin polymer [34]. The low dough strengths of composite doughs can be explained by the high content of dietary fibre in non-wheat flours, which hindered formation of gluten viscoelastic networks. The ratio of maximum resistance to extension/extensibility (MR/E) increased when incubation time was extended from 45 to 90 min (Table 3). However, when incubation time was further extended to 135 min, MR/E of WHE-NFM, WHE-SFM and WHE-MFM decreased by between 21 and 30%. In contrast, MR/E of wheat and WHE-NAM decreased by smaller margins of about 10%. The MR/E of WHE-SAM did not change whereas that of WHE-MAM increased by 17% when incubation time was prolonged to 135 min. The positive effect of amaranth on MR/E in composite doughs was attributed to the interaction of amaranth albumins with glutenin polymers [11,12]. Dough with high MR/E value has high strength relative to extensibility and, up to a certain limit, is expected to give bread with a high volume.

### 3.3. Physical and Textural Properties of Breads

**Figure 1:**
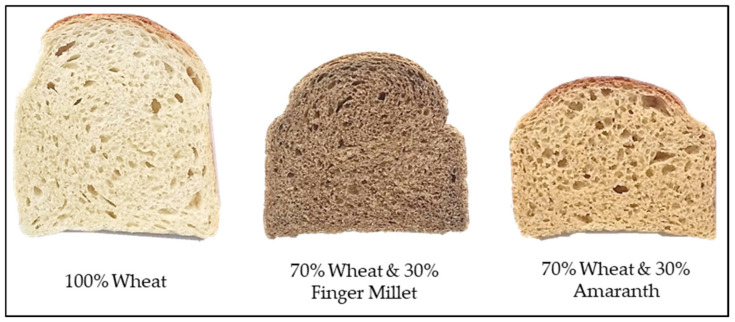
Cut-through sections of breads produced using 100% wheat flour, 70% wheat + 30% native finger millet flour and 70% wheat + 30% amaranth flour.

Substantial differences regarding bread volume and colour, as well as the crumb structure, were visible depending on the type of composite flour used (Figure 1). The weights of breads ranged between 335–344 g and the volumes ranged between 1110–1448 cm^3^ (Table 4). The specific volumes of composite breads were lower by between 9–28% compared to wheat bread. Specific volume is an important quality parameter of bread because it is largely associated with the appearance of bread. The distinctive high volume of bread is attributed to gluten, which influences the gas retention properties of fermenting dough [34]. Substitution of wheat with gluten-free flour reduces bread volume due to the combined effects of gluten dilution and disruption of gluten matrix by non-starch polysaccharides [5,6]. Partial dehydration of gluten due to competition with fibre for hydration is responsible for the structural changes of gluten matrix and collapse of the gluten polymeric network [4]. In addition, dietary fibre disrupts formation and physical properties of gluten network through interactions of its reactive components (especially ferulic acid monomers) with gluten proteins [35]. The high water-holding capacity of dietary fibre also reduces the amount of steam generated, which results in decreased loaf volume.

The WHE-NAM and WHE-MAM breads had higher (*p* < 0.05) specific volumes and lower (*p* < 0.05) crumb firmness than the other composite breads (Table 4). Crumb firmness is inversely related to specific volume and breads with low specific volumes tend to have high crumb firmness because of their compact and closed crumb structure [5,8]. Crumb firmness in composite bread is influenced by the botanical origin of non-wheat flour and degree of wheat substitution [5,7,8]. Dietary fibre is the main cause of high crumb firmness in composite breads, since it strengthens the walls which surround air bubbles in the crumb [6,33]. The low crumb firmness of WHE-NAM and WHE-MAM breads could be attributed to formation of stable disulphide linkages between amaranth albumins and wheat glutenin polymers [11]. Silva-Sánchez et al. [12] found that albumin isolates (1–3% *w*/*w*) in a bread recipe improves its volume and crumb texture. However, the low specific volume and high crumb firmness of WHE-SAM bread indicate absence of albumin–gluten interactions in its dough probably because albumins lost their functionality through denaturation during steaming. Steaming decreases, whereas germination increases, the content of albumins in amaranth [36]. Although drying (90 °C) germinated amaranth decreases the amount of water-soluble proteins, the net amount is still higher than in native or steamed amaranth [36] and contributes to low crumb firmness.

The impact of gluten dilution and interference during gluten network formation in composite formulas was evident in the poorer crumb structure of composite breads compared to wheat bread. Composite breads had lower (*p* < 0.05) crumb cohesiveness, resilience and springiness than wheat bread (Table 4). Crumb chewiness (product of crumb firmness, cohesiveness and springiness), which indicates the energy required to chew bread into a state suitable for swallowing, closely imitated crumb firmness rather than cohesiveness or springiness of the breads. Crumb chewiness of WHE-NAM and WHE-MAM breads were not significantly different (*p* > 0.05) to WHE bread. In addition, WHE-NAM and WHE-MAM breads were more cohesive and resilient than WHE-SAM or WHE-NFM, WHE-SFM and WHE-MFM breads. The better crumb texture of WHE-NAM and WHE-MAM breads compared to the other composite breads was attributed to the presence of functionally active albumins in amaranth, as explained before.

The change in crumb lightness (ΔL*) of composite breads was closely related to the colours of native finger millet and amaranth flours. The lightness index (L*) of wheat flour was 81 ± 3. Finger millet has darker (*t*-test, *p* < 0.001) seed coat pigmentation (L* = 64 ± 3) than amaranth (L* = 73 ± 2). Consequently, WHE-NFM, WHE-SFM and WHE-MFM breads were darker (*p* < 0.05) compared to WHE-NAM, WHE-SAM and WHE-MAM breads (Table 4). Crumb lightness of WHE-MFM or WHE-SFM breads was not significantly different (*p* > 0.05) to WHE-NFM bread. In contrast, WHE-SAM and WHE-MAM breads had darker crumbs than WHE-NAM bread. The darker crumbs of WHE-SAM and WHE-MAM breads may be associated with Maillard and caramelization reactions in the crumb arising from the high contents of free sugars in steamed or malted amaranth. Due to the potential adverse health effects of Maillard reaction products, such as acrylamide [37], the development of WHE-SAM and WHE-MAM breads must be further optimized.

### 3.4. Nutrient Qualities of Bread

The WHE-NAM, WHE-SAM and WHE-MAM breads contained higher (*p* < 0.05) monosaccharide but lower (*p* < 0.05) disaccharide contents than wheat or WHE-NFM, WHE-SFM and WHE-MFM breads (Table 5). Trisaccharides were not present whereas the contents of sugar alcohols were less than 25 mg/100 g in all breads. The total sugar contents of the breads were cumulative values of the sugars naturally present in the flours (Table 1), sugar used in the breadmaking recipe and sugars derived from diastatic activity on damaged starch. The total content of free sugars (i.e., monosaccharides and disaccharides) increased from 2924 mg/100 g in WHE-NFM to 3160 mg/100 g in WHE-SFM bread. By contrast, it decreased from 3862 mg/100 g in WHE-NAM to 3762 mg/100 g in WHE-SAM bread. The total content of free sugars was higher in WHE-MFM (3666 mg/100 g) and WHE-MAM (4799 mg/100 g) than in WHE-NFM (2924 mg/100 g) and WHE-NAM (3862 mg/100 g), respectively.

There were no significant differences (*p* > 0.05) in the starch and protein contents and digestibilities of the different breads (Table 5). The WHE-NAM, WHE-SAM and WHE-MAM breads had higher (*p* < 0.05) lipid contents than wheat or WHE-NFM, WHE-SFM and WHE-MFM breads due to the higher lipid content of amaranth (Table 1). Composite breads had higher (*p* < 0.05) ash, phytate and phenolic acid contents than wheat bread due to the inherently higher amounts of these compounds in whole-milled finger millet and amaranth (Table 1). Composite breads had higher arabinoxylan and insoluble and total dietary fibre contents but lower soluble dietary fibre contents than wheat bread, which originated from the different dietary fibre composition of amaranth and finger millet compared to wheat. Since regular consumption of dietary fibre, in particular arabinoxylans, is recommended for a healthy diet [38,39], the composite breads had a higher nutritional value than the wheat breads. The WHE-NAM, WHE-SAM and WHE-MAM breads had lower arabinoxylan molecular weights than wheat or WHE-NFM, WHE-SFM and WHE-MFM. Higher molar mass arabinoxylans are generally associated with better nutraceutical properties, due to increased viscosity in the intestine [39], indicating lower nutritional value for amaranth composite breads.

## 4. Conclusions

The impact of native, steamed or malted finger millet and amaranth on dough and bread quality was investigated. While the properties of wheat dough are primarily determined by gluten, in the composite doughs, dietary fibre and protein from finger millet and amaranth were found to have an effect as well. In general, doughs containing finger millet had poorer rheological qualities than doughs containing amaranth. Amongst composite doughs, WHE-NAM and WHE-MAM had the best rheological properties, which translated to breads with high volume and good crumb texture. The suitability of amaranth for making composite bread was attributed to its albumin fraction that forms stable disulphide linkages to wheat glutenin, whereas the poor baking quality of finger millet was attributed to its dietary fibre fraction by hindering the formation of gluten viscoelastic networks. The addition of finger millet or amaranth did not change the starch and protein contents or digestibilities of bread. However, they improved the dietary fibre, ash and phenolic acid contents of bread. This study shows that the type of grain and its modification influences the quality of composite dough and bread. Based on the results, the use of native or malted amaranth appears as a promising approach for the production of high quality breads with the added benefit of significantly higher dietary fibre content than the reference wheat bread. There is a need for further optimization to increase the amount of amaranth that can be added to composite bread without quality deterioration. This could make an increase in bread protein content possible. Furthermore, future studies should determine the acrylamide content when using malted amaranth, to ensure consumer safety. With respect to finger millet, steaming and malting were not suitable to improve its breadmaking potential. Hence, other techniques of flour modification should be explored in future studies to enlarge the range of applications for this crop.

## Figures and Tables

**Table 1:** Nutrient composition (based on dry weight) and quality of wheat, finger millet and amaranth flour.

Nutrient	Wheat	Finger Millet			Amaranth		
		Native	Steamed	Malted	Native	Steamed	Malted
Monosaccharides (mg/100 g) *	53 ± 1 ^g^	77 ± 0 ^e^	219 ± 1 ^d^	1257 ± 0 ^a^	65 ± 0 ^f^	282 ± 1 ^c^	1030 ± 0 ^b^
Disaccharides (mg/100 g) **	710 ± 0 ^c^	692 ± 1 ^d^	460 ± 0 ^f^	54 ± 0 ^g^	784 ± 0 ^b^	593 ± 1 ^e^	817 ± 0 ^a^
Trisaccharides (mg/100 g) ***	104 ± 1 ^d^	82 ± 1 ^e^	71 ± 1 ^f^	nd	760 ± 0 ^a^	451 ± 0 ^b^	226 ± 0 ^c^
Sugar alcohols (mg/100 g) ****	12 ± 0 ^f^	30 ± 0 ^b^	15 ± 0 ^d^	7 ± 0 ^g^	14 ± 0 ^e^	18 ± 0 ^c^	63 ± 1 ^a^
Total starch (g/100 g)	73 ± 0 ^bc^	82 ± 0 ^a^	83 ± 1 ^a^	79 ± 1 ^ab^	69 ± 1 ^c^	61 ± 5 ^d^	58 ± 0 ^d^
Digestible starch (% of total starch)	88 ± 2 ^b^	84 ± 1 ^b^	86 ± 3 ^b^	88 ± 1 ^b^	97 ± 1 ^a^	97 ± 0 ^a^	98 ± 2 ^a^
Insoluble dietary fibre (g/100 g)	2.3 ± 0.2 ^d^	12 ± 0 ^a^	12 ± 0 ^ab^	11 ± 0 ^ab^	7.6 ± 0.2 ^c^	8.5 ± 0.9 ^c^	10 ± 2 ^b^
Soluble dietary fibre (g/100 g)	1.3 ± 0.3 ^a^	0.6 ± 0.2 ^b^	0.6 ± 0.5 ^b^	0.8 ± 0.3 ^b^	0.7 ± 0.3 ^b^	1.0 ± 0.1 ^ab^	1.0 ± 0.0 ^ab^
Total dietary fibre (g/100 g)	3.6 ± 0.3 ^c^	13 ± 0 ^a^	12 ± 1 ^a^	12 ± 0 ^a^	8.3 ± 0.4 ^b^	9.5 ± 0.9 ^b^	11 ± 2 ^a^
Arabinoxylan (mg/100 g)	1312 ± 0 ^d^	1555 ± 2 ^b^	1455 ± 1 ^c^	2017 ± 3 ^a^	1061 ± 2 ^g^	1184 ± 0 ^f^	1228 ± 1 ^e^
Arabinoxylan molecular weight (kDa)	195 ± 25 ^a^	177 ± 16 ^a^	189 ± 17 ^a^	166 ± 8 ^a^	99 ± 4 ^b^	139 ± 15 ^ab^	93 ± 10 ^b^
Total protein (g/100 g)	15 ± 1 ^a^	11 ± 0 ^bc^	9 ± 0 ^c^	9 ± 1 ^c^	14 ± 2 ^ab^	16 ± 1 ^a^	18 ± 2 ^a^
In vitro protein digestibility (% of total protein)	80 ± 4 ^b^	88 ± 2 ^ab^	79 ± 2 ^b^	80 ± 1 ^b^	87 ± 4 ^ab^	92 ± 0 ^a^	93 ± 1 ^a^
Lipid (g/100 g)	1.8 ± 0.1 ^c^	1.3 ± 0.0 ^d^	1.6 ± 0.1 ^cd^	1.4 ± 0.3 ^cd^	8.0 ± 0.1 ^ab^	7.6 ± 0.3 ^b^	8.3 ± 0.1 ^a^
Ash (g/100 g)	0.8 ± 0.0 ^c^	3.9 ± 0.0 ^a^	3.4 ± 0.5 ^ab^	3.1 ± 0.0 ^b^	2.9 ± 0.1 ^b^	2.8 ± 0.0 ^b^	3.2 ± 0.0 ^b^
Phytate (mg/100 g)	621 ± 69 ^c^	1260 ± 133 ^c^	1087 ± 37 ^c^	1144 ± 311 ^c^	1366 ± 310 ^bc^	2062 ± 107 ^ab^	2209 ± 176 ^a^
Total phenolic content (mg GAE/100 g)	103 ± 5 ^c^	162 ± 2 ^a^	162 ± 2 ^a^	142 ± 6 ^b^	39 ± 0 ^f^	68 ± 4 ^e^	85 ± 0 ^d^

* Glucose and fructose; ** sucrose and maltose; *** raffinose; **** sorbitol and mannitol. nd: not detected. Values presented as mean ± standard deviation; *n* = 3. Values in the same row with different superscript letters are significantly different at *p* < 0.05.

**Table 2:** Enzyme activity of flours and farinogram properties of doughs.

Flour	α-Amylase Activity (CU/g)	WAC (%)	DDT (min)	Stability (min)	DS (FU)	FQN (mm)
WHE	0.6 ± 0.1	59 ± 0.2 ^e^	1.9 ± 0.2 ^b^	3.6 ± 0.3 ^c^	76 ± 4 ^d^	40 ± 3 ^d^
WHE-NFM	0.6 ± 0.0	58 ± 0.1 ^e^	1.8 ± 0.4 ^b^	5.5 ± 0.1 ^a^	122 ± 2 ^c^	55 ± 1 ^c^
WHE-SFM	0.4 ± 0.2	60 ± 0.0 ^d^	1.5 ± 0.1 ^b^	5.2 ± 0.3 ^ab^	113 ± 7 ^c^	56 ± 1 ^c^
WHE-MFM	0.7 ± 0.0	61 ± 0.2 ^bc^	1.4 ± 0.3 ^b^	2.5 ± 0.0 ^d^	168 ± 11 ^b^	32 ± 1 ^e^
WHE-NAM	0.5 ± 0.1	61 ± 0.1 ^c^	3.8 ± 0.4 ^a^	5.0 ± 0.7 ^ab^	114 ± 6 ^c^	70 ± 1 ^a^
WHE-SAM	0.4 ± 0.0	63 ± 0.3 ^a^	3.8 ± 0.3 ^a^	4.1 ± 0.1 ^bc^	132 ± 8 ^c^	66 ± 4 ^ab^
WHE-MAM	0.6 ± 0.2	62 ± 0.1 ^b^	4.2 ± 0.1 ^a^	4.2 ± 0.1 ^bc^	193 ± 1 ^a^	61 ± 1 ^bc^

CU/g: ceralpha units/g; WHE: wheat; NFM: native finger millet; SFM: steamed finger millet; MFM: malted finger millet; NAM: native amaranth; SAM: steamed amaranth; MAM: malted amaranth; WAC: water absorption capacity; DDT: dough development time; DS: degree of softening; FQN: farinograph quality number; FU: farinograph units. Values presented as mean ± standard deviation; *n* = 3. Values in the same column followed by the same lower-case letter are not significantly different from each other (*p* < 0.05). Values in the same column not followed by lower-case letter are not significantly different from each other (*p* < 0.05).

**Table 3:** Extensogram properties of dough.

Dough	45 min				90 min				135 min			
	Energy (cm^2^)	E (mm)	MR (EU)	MR/E	Energy (cm^2^)	E (mm)	MR (EU)	MR/E	Energy (cm^2^)	E (mm)	MR (EU)	MR/E
WHE	127 ± 11 ^a^	167 ± 10 ^a^	626 ± 62 ^a^	3.9 ± 0.5 ^a^	102 ± 6 ^a^	139 ± 3 ^a^	661 ± 33 ^a^	4.8 ± 0.2 ^b^	70 ± 8 ^a^	122 ± 10 ^a^	523 ± 22 ^a^	4.3 ± 0.3 ^ab^
WHE-NFM	54 ± 6 ^b^	93 ± 1 ^b^	446 ± 43 ^b^	4.8 ± 0.4 ^a^	38 ± 1 ^b^	74 ± 3 ^c^	432 ± 18 ^b^	5.9 ± 0.1 ^a^	16 ± 2 ^b^	68 ± 7 ^b^	189 ± 14 ^d^	2.8 ± 0.1 ^cd^
WHE-SFM	45 ± 0 ^bc^	85 ± 0 ^b^	405 ± 8 ^b^	4.8 ± 0.1 ^a^	35 ± 1 ^bc^	70 ± 1 ^c^	432 ± 17 ^b^	6.2 ± 0.3 ^a^	25 ± 3 ^b^	66 ± 5 ^b^	321 ± 16 ^b^	4.9 ± 0.1 ^a^
WHE-MFM	44 ± 3 ^bc^	88 ± 2 ^b^	383 ± 36 ^b^	4.4 ± 0.6 ^a^	29 ± 6 ^bc^	72 ± 4 ^c^	330 ± 46 ^cd^	4.6 ± 0.4 ^b^	17 ± 5 ^b^	63 ± 7 ^b^	204 ± 52 ^cd^	3.2 ± 0.4 ^c^
WHE-NAM	43 ± 3 ^bc^	89 ± 4 ^b^	379 ± 12 ^b^	4.3 ± 0.0 ^a^	39 ± 1 ^b^	79 ± 5 ^bc^	410 ± 18 ^bc^	5.3 ± 0.5 ^ab^	32 ± 3 ^b^	76 ± 1 ^b^	352 ± 33 ^b^	4.7 ± 0.5 ^a^
WHE-SAM	27 ± 3 ^c^	99 ± 8 ^b^	198 ± 4 ^c^	2.0 ± 0.1 ^b^	22 ± 0 ^c^	89 ± 1 ^b^	183 ± 3 ^e^	2.1 ± 0.1 ^c^	20 ± 1 ^b^	84 ± 2 ^b^	178 ± 4 ^d^	2.1 ± 0.0 ^d^
WHE-MAM	28 ± 1 ^c^	104 ± 0 ^b^	199 ± 9 ^c^	1.9 ± 0.1 ^b^	29 ± 2 ^bc^	88 ± 2 ^b^	252 ± 7 ^de^	2.9 ± 0.0 ^c^	31 ± 2 ^b^	84 ± 3 ^b^	285 ± 6 ^bc^	3.4 ± 0.0 ^bc^

WHE: wheat; NFM: native finger millet; SFM: steamed finger millet; MFM: malted finger millet; NAM: native amaranth; SAM: steamed amaranth; MAM: malted amaranth; E: extensibility; MR: maximum resistance to extension; EU: extensograph units. Values presented as mean ± standard deviation; *n* = 3. Values in the same column followed by the same lower-case letter are not significantly different from each other (*p* < 0.05).

**Table 4:** Physical and textural properties of bread.

Bread	Weight (g)	Volume (cm^3^)	Specific Volume (cm^3^/g)	Firmness (N)	Cohesiveness **	Resilience **	Springiness (%)	Chewiness (N)	ΔL*
WHE	341 ± 2 ^ab^	1448 ± 58 ^a^	4.3 ± 0.2 ^a^	3.0 ± 0.5 ^d^	0.74 ± 0.02 ^a^	0.31 ± 0.02 ^a^	91 ± 1 ^a^	2.0 ± 0.3 ^e^	-
WHE-NFM	335 ± 2 ^c^	1135 ± 30 ^d^	3.4 ± 0.1 ^cd^	7.2 ± 1.0 ^bc^	0.56 ± 0.02 ^d^	0.22 ± 0.01 ^de^	88 ± 1 ^b^	3.5 ± 0.5 ^bc^	−20 ± 1 ^c^
WHE-SFM	340 ± 1 ^ab^	1135 ± 70 ^d^	3.3 ± 0.1 ^cd^	8.8 ± 0.8 ^b^	0.57 ± 0.04 ^d^	0.23 ± 0.02 ^cde^	86 ± 1 ^bc^	4.3 ± 0.6 ^b^	−19 ± 1 ^c^
WHE-MFM	344 ± 1 ^a^	1070 ± 26 ^d^	3.1 ± 0.1 ^d^	6.6 ± 0.5 ^c^	0.55 ± 0.01 ^d^	0.22 ± 0.01 ^e^	86 ± 2 ^bc^	3.1 ± 0.3 ^cd^	−21 ± 1 ^c^
WHE-NAM	339 ± 2 ^b^	1240 ± 28 ^c^	3.7 ± 0.1 ^bc^	4.2 ± 0.4 ^d^	0.67 ± 0.01 ^bc^	0.27 ± 0.01 ^b^	86 ± 1 ^bc^	2.4 ± 0.2 ^de^	−8 ± 3 ^a^
WHE-SAM	344 ± 2 ^a^	1110 ± 42 ^d^	3.2 ± 0.2 ^d^	10.7 ± 1.4 ^a^	0.63 ± 0.03 ^c^	0.25 ± 0.01 ^bcd^	86 ± 1 ^bc^	5.8 ± 0.6 ^a^	−10 ± 4 ^ab^
WHE-MAM	344 ± 2 ^a^	1350 ± 26 ^b^	3.9 ± 0.1 ^b^	2.7 ± 0.2 ^d^	0.68 ± 0.02 ^b^	0.26 ± 0.01 ^bc^	85 ± 1 ^c^	1.6 ± 0.1 ^e^	−13 ± 2 ^b^

WHE: wheat; NFM: native finger millet; SFM: steamed finger millet; MFM: malted finger millet; NAM: native amaranth; SAM: steamed amaranth; MAM: malted amaranth. ΔL* = L*_wheat bread_ − L*_composite bread._ ** Dimensionless terms. Values presented as mean ± standard deviation; *n* = 3. Values in the same column followed by the same lower-case letter are not significantly different from each other (*p* < 0.05).

**Table 5:** Nutrient composition (based on dry weight) and quality of bread.

Nutrient	WHE	WHE-NFM	WHE-SFM	WHE-MFM	WHE-NAM	WHE-SAM	WHE-MAM
Monosaccharides (mg/100 g) *	1047 ± 1 ^g^	1347 ± 0 ^f^	1532 ± 1 ^e^	1715 ± 2 ^d^	3275 ± 1 ^b^	3253 ± 2 ^c^	4085 ± 1 ^a^
Disaccharides (mg/100 g) **	2877 ± 2 ^a^	1577 ± 0 ^d^	1628 ± 1 ^c^	1951 ± 1 ^b^	587 ± 0 ^f^	509 ± 0 ^g^	714 ± 1 ^e^
Sugar alcohols (mg/100 g) ***	23 ± 0 ^b^	25 ± 0 ^a^	19 ± 0 ^c^	15 ± 0 ^e^	11 ± 0 ^g^	13 ± 0 ^f^	17 ± 0 ^d^
Total starch (g/100 g)	78 ± 1	77 ± 1	78 ± 3	78 ± 0	76 ± 0	76 ± 0	76 ± 0
Digestible starch (% of total starch)	96 ± 4	96 ± 3	95 ± 2	95 ± 2	98 ± 2	98 ± 1	98 ± 1
Insoluble dietary fibre (g/100 g)	2.7 ± 0.4 ^c^	5.4 ± 0.2 ^a^	5.5 ± 0.1 ^a^	5.5 ± 0.2 ^a^	4.4 ± 0.4 ^b^	4.1 ± 0.3 ^b^	4.5 ± 0.3 ^b^
Soluble dietary fibre (g/100 g)	1.8 ± 0.2 ^a^	1.7 ± 0.3 ^ab^	1.3 ± 0.1 ^b^	1.4 ± 0.2 ^b^	1.5 ± 0.2 ^ab^	1.6 ± 0.1 ^ab^	1.5 ± 0.2 ^ab^
Total dietary fibre (g/100 g)	4.5 ± 0.4 ^c^	7.1 ± 0.3 ^a^	6.8 ± 0.2 ^a^	6.9 ± 0.2 ^a^	6.0 ± 0.5 ^b^	5.7 ± 0.2 ^b^	6.0 ± 0.1 ^b^
Arabinoxylan (mg/100 g)	1363 ± 0 ^g^	1375 ± 0 ^f^	1413 ± 1 ^d^	1480 ± 1 ^b^	1497 ± 0 ^a^	1410 ± 0 ^e^	1441 ± 1 ^c^
Arabinoxylan molecular weight (kDa)	153 ± 5 ^a^	120 ± 16 ^bc^	134 ± 10 ^ab^	119 ± 6 ^bc^	91 ± 7 ^cd^	90 ± 0 ^cd^	85 ± 6 ^d^
Total protein (g/100 g)	14 ± 1	12 ± 0	12 ± 0	15 ± 2	15 ± 1	14 ± 2	15 ± 1
In vitro protein digestibility (% of total protein)	91 ± 3	89 ± 1	83 ± 5	87 ± 1	89 ± 2	84 ± 1	86 ± 2
Lipid (g/100 g)	2.1 ± 0.5 ^ab^	1.4 ± 0.3 ^b^	1.2 ± 0.2 ^b^	1.4 ± 0.2 ^b^	2.7 ± 0.3 ^a^	2.9 ± 0.0 ^a^	2.9 ± 0.2 ^a^
Ash (g/100 g)	1.6 ± 0.2 ^c^	2.5 ± 0.1 ^a^	2.4 ± 0.0 ^a^	2.2 ± 0.0 ^ab^	2.2 ± 0.0 ^ab^	2.2 ± 0.0 ^ab^	1.9 ± 0.1 ^bc^
Phytate (mg/100 g)	170 ± 36 ^b^	609 ± 141 ^a^	551 ± 7 ^a^	598 ± 8 ^a^	668 ± 15 ^a^	698 ± 16 ^a^	669 ± 44 ^a^
Total phenolic content (mg GAE/100 g)	59 ± 3 ^d^	88 ± 7 ^bc^	84 ± 9 ^bc^	94 ± 11 ^b^	60 ± 3 ^d^	74 ± 9 ^cd^	122 ± 10 ^a^

* Glucose and fructose; ** sucrose and maltose; *** sorbitol and mannitol. WHE: wheat; NFM: native finger millet; SFM: steamed finger millet; MFM: malted finger millet; NAM: native amaranth; SAM: steamed amaranth; MAM: malted amaranth; GAE: gallic acid equivalent. Values presented as mean ± standard deviation; *n* = 3. Values in the same row followed by the same lower-case letter are not significantly different from each other (*p* < 0.05). Values in the same row not followed by a lower-case letter are not significantly different from each other (*p* < 0.05).

## Data Availability

Data is contained within the article.
